# A generic screening platform for inhibitors of virus induced cell fusion using cellular electrical impedance

**DOI:** 10.1038/srep22791

**Published:** 2016-03-15

**Authors:** Daniel Watterson, Jodie Robinson, Keith J. Chappell, Mark S. Butler, David J. Edwards, Scott R. Fry, Imogen M. Bermingham, Matthew A. Cooper, Paul R. Young

**Affiliations:** 1 School of Chemistry and Molecular Biosciences, University of Queensland, Brisbane, Queensland 4072, Australia; 2 Division of Chemistry and Structural Biology, Institute for Molecular Bioscience, University of Queensland, Brisbane, Queensland 4072, Australia; 3 Australian Infectious Diseases Research Centre, University of Queensland, Brisbane, Queensland 4072, Australia

## Abstract

Fusion of the viral envelope with host cell membranes is an essential step in the life cycle of all enveloped viruses. Despite such a clear target for antiviral drug development, few anti-fusion drugs have progressed to market. One significant hurdle is the absence of a generic, high-throughput, reproducible fusion assay. Here we report that real time, label-free measurement of cellular electrical impedance can quantify cell-cell fusion mediated by either individually expressed recombinant viral fusion proteins, or native virus infection. We validated this approach for all three classes of viral fusion and demonstrated utility in quantifying fusion inhibition using antibodies and small molecule inhibitors specific for dengue virus and respiratory syncytial virus.

Although the fusion of two lipid bilayers is a thermodynamically favorable reaction, the kinetic barrier to their merger is substantial^1^. Viruses overcome this energy barrier by employing fusion proteins that, once triggered, drive the fusion process by a series of coordinated conformational changes from a metastable pre-fusion configuration to its final, most energetically stable post-fusion form^2^. There are currently three recognized classes of viral fusion proteins distinguished by their molecular architecture, referred to as classes I-III (reviewed in^3^,^4^,^5^). Despite significant differences in structure, a common fusion mechanism has been proposed, where either receptor binding or low pH within the cellular endosome, triggers the formation of an elongated intermediate fusion protein that inserts a hydrophobic fusion peptide (FP) into the target host membrane. Subsequent collapse of these intermediates into a low energy hairpin-like structure then provides the driving force required for membrane fusion.

These mechanistic insights have been derived from structural studies of pre- and post-fusion forms of enveloped virus fusion proteins representative of each of the three fusion classes and have provided the basis for rational design of fusion inhibitors that can inhibit key steps in this process^6^,^7^. Viral entry inhibitors offer several advantages over compounds that target essential viral enzyme activity, including virus inactivation prior to cell infection, and potentially improved bioavailability given that anti-fusion drugs are not dependent on cellular entry.

The prototype fusion inhibitor, enfuvirtide, targets the HIV protein responsible for virus and host cell membrane fusion, gp41^8^,^9^,^10^. The development of enfuvirtide and its analogs was only possible with intimate knowledge of the gp41 structure and the development of a range of cell and *in vitro* based fusion assays that were designed *specifically for HIV*. These types of assays are not always readily adaptable to other viruses, and are time consuming and laborious to perform. Such limitations have impeded the development of second generation, small molecule fusion inhibitors. Here we report a real time, label-free, cell based assay that uses cellular electrical impedance (CEI) as a quantifiable measure of viral induced cell fusion.

Label-free cell based assays are gaining acceptance as non-invasive real time transducers of cellular processes^11^,^12^,^13^. Electrical impedance assays are one such approach suitable for plate-based screening of live cells^14^,^15^. Cell membranes are essentially non-conductive, when viewed in comparison to the highly conductive cytoplasm and biological buffers with high electrolyte content. Consequently, when a low frequency (kHz) alternating current is applied to a cell monolayer grown on electrodes in a plate well, the cell membrane capacitance affects the cell impedance, which is dominated by ions flowing around or between cells; termed the extra-cellular current. In contrast, if measurements are acquired at high frequencies (MHz), the electric field alternates polarity so rapidly that there is not sufficient time for ions to accrue at the cell membrane surface, resulting in the shielding of the interior of the cell from the externally applied electric field. Essentially, the cell membrane appears electrically transparent, with ionic currents that appear to flow across cell membranes being termed trans-cellular current^16^,^17^,^18^. Commercially available impedance systems exploit this frequency-variant behaviour of cell impedance and report the ratio of current measured at the different frequencies to monitor cell morphology and intracellular process. This is normally done using inter-digitated gold electrodes fabricated on the bottom of cell culture plates. Voltages are maintained at a low level to prevent conversion of ionic current to electrical current at the electrode-buffer interface so that an ionic double layer builds up at the electrodes at low frequencies resulting in electrode polarization. This, in turn, results in an increased impedance magnitude and lagging impedance phase, or capacitive behaviour^19^ that can be measured in cells sedimented on, adhering to, or growing on coplanar electrodes^20^,^21^,^22^. The relative change in measured electrical impedance is then most often quoted as a ‘cell index’ in commercial systems.

The application of electrical impedance for monitoring and understanding cell-virus interactions has been limited to detection of virus binding using an immunoassay^23^, and the observation of virus-induced cytopathic effects^24^,^25^,^26^. These are very different phenomena to virus-induced fusion; the mode of action to be selected in our search for new inhibitors within this nascent class of anti-viral drugs. To our knowledge, the work we report here is the first to measure virus-induced cell fusion in real time using electrical impedance monitoring. Furthermore, we show that this approach to quantifying virus-mediated cell membrane fusion is applicable to all three enveloped virus fusion classes identified to date, and can be translated to a high-throughput format. The utility of the assay platform was confirmed using fusion inhibitory monoclonal antibodies and a panel of small molecule fusion inhibitors. This assay could be readily adapted to a wide variety of viral pathogens and provides a non-invasive real-time option for plate-based high-throughput screening of live cells^14^,^15^.

## Results

### Cellular electrical impedance (CEI) is a quantifiable measure of virus-induced cell membrane fusion

**Figure 1:**
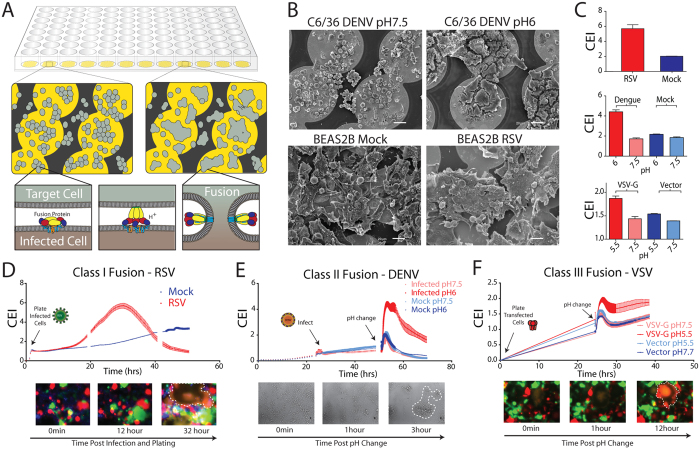
Cellular electrical impedance as a measure of viral induced cell fusion. (**A**) Assay schematic. Cells are grown in microtitre plates (96- or 384-well) with embedded gold microelectrodes. Viral fusion proteins expressed on the surface of infected or transfected cells are triggered to undergo a conformational change following receptor binding or exposure to low pH depending on viral fusion class. This conformational change initially involves the formation of a short-lived, elongated intermediate that collapses into a trimeric hairpin, which drives the merger of neighboring cell membranes (dengue virus E protein mediated membrane fusion is schematically shown in the lower inset). (**B**) SEM of cells growing on microelectrodes. Cells were fixed and imaged when infected cells showed maximum cell impedance. The large syncytia that accompany cell fusion are clearly visible in both DENV and RSV infected cells (C6/36 insect cells and BEAS2B human bronchial airway epithelial cells, respectively). (**C**) Maximum cell index, CI (bars represent SE; N = 3), following infection of cells by RSV and DENV or transient expression of recombinant VSV G. show significant membrane fusion for all three fusion classes. (**D–F**) Raw, real-time output of impedance measurements over the time course of the fusion assay reveals quantitative fusion detection that correlates with live cell microscopy (lower panels) for all three classes of viral fusion proteins. Microscopy based assays were performed with C6/36 cells for DENV and imaged in bright field, while RSV and VSV fusion were visualized using fluorescent microcopy of a mixed population of stable COS-7 cells expressing GFP and mCherry, fusion is visualised as an overlap of the GFP and mCherry signals. Measurements were performed on infected (RSV and DENV) cells and cells transiently expressing recombinant VSV-G. Maximal cell impedance for DENV and VSV G was only observed following treatment of cells with low pH.

To demonstrate practical advantages and broad potential of the assay we assessed cellular fusion induced by all three viral fusion classes, represented by respiratory syncytial virus (RSV, class I), dengue virus (DENV, class II) and vesicular stomatitis virus (VSV, class III). A simplified overview of the assay design is shown in Fig. 1A. Cells are first seeded into microtiter plates, containing embedded microelectrodes and subsequently infected with virus or transfected with expression plasmids encoding viral fusion proteins. Fusion is then triggered by the addition of acidified media, which mimics the low pH environment of the endosome where viral entry occurs for some viruses (DENV and VSV), or once viral fusion glycoproteins are transported to the cell surface where they can interact with receptor molecules on a neighboring cell (RSV). Fusion protein mediated cell fusion was then monitored in real time as a function of cellular electrical impedance.

In all three viral fusion classes, cell syncytia formation, observed by live cell microscopy, was accompanied by significant increases in cell impedance (Fig. 1D–F and Supplementary Fig. S1) suggesting that the micro-environment surrounding the embedded electrodes in contact with the cell was becoming less conductive to ion flow. Scanning electron microscopy analysis of infected cells fixed at the time of maximum CEI signal clearly showed the gross morphological changes of the cell monolayer that accompanied cell fusion (Fig. 1B). The fusion of cells substantially decreased the extracellular space directly above the embedded electrodes, which in turn generated the significant increase in impedance measurement observed. The CEI signature appeared to mirror the formation of syncytia (see Supplementary Fig. S2) and confirmed that fusion could be monitored in real time, which may be exploited in future studies to examine fusion kinetics.

After reaching maximal impedance, the fusion signature was seen to decrease for all fusion classes, which may be attributable to the degradation of the cell monolayer and the instability of the multinucleated cell membrane. However, due to the real time nature of the label-free CEI measurement, it was simply a matter of identifying the optimal fusion time point prior to monolayer disruption, which can then be used as a set time point to probe the fusion process and discover and characterize molecules that interfere with this process (Fig. 1C).

After validating the practicality of CEI as a method for quantifying virus driven cell fusion, we sought to examine the utility of the assay to characterize fusion inhibiting therapeutic candidates for DENV and RSV; viral pathogens that impact human health worldwide.

Indeed, in the case of DENV, a reproducible fusion assay has long been problematic. This is likely due to the specific requirements of DENV driven cell fusion, as viral induced syncytia form only when mosquito cells are used, unless the lipid components of the mammalian plasma membrane are altered artificially^27^. In contrast to traditional reporter based fusion assays, a label-free approach is ideally suited to cell systems that do not have reporter reagents readily available. However for RSV, which forms large syncytia within an *in vivo* context, a number of fusion assay systems have been previously developed^28^,^29^,^30^, which allow a direct comparative analysis with our label-free fusion assay system.

### Antibody mediated neutralization of dengue virus can be quantified by CEI

**Figure 2:**
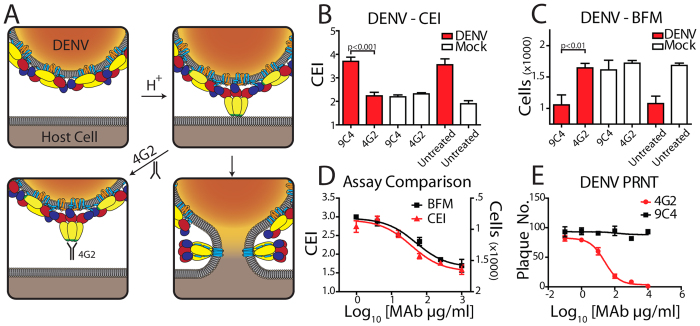
Label-free quantification of dengue fusion inhibition by a monoclonal antibody. (**A**) Schematic of the mode of action for the DENV neutralizing monoclonal antibody 4G2, which binds to the fusion peptide, FP. After acidification, molecular rearrangement of the E protein exposes the hydrophobic FP (green), which associates with the host cell membrane. 4G2 binding to the exposed FP prevents membrane insertion and therefore blocks membrane fusion. (**B**) Measurement of the anti-fusion effects of 4G2 using cellular impedance. Cells were infected at an MOI of 2 or mock infected. Twenty-four hour post infection the culture media was replaced with fresh media at pH 6 and the CI recorded. The cell impedance measurement at 1.5 h post media change is shown. The presence of 4G2 (500 μg/ml) within the low pH media was sufficient to completely abrogate the fusion signal. An isotype matched antibody control, 9C4 (specific for the RSV F protein) had no significant effect. (**C**) Parallel fusion assay setup to (B) was observed using live cell bright field microscopy. Similar anti-fusion effects of 4G2 were observed, with fusion extent inversely proportional to cell number. (**D**) A dose response analysis of the inhibitory effect of 4G2 using both cell impedance and live cell microscopy revealed near identical activity profiles. The CEI measurement is indicated on the left axis and BFM measurements are plotted on an inverted right axis to allow direct comparison of the dose response curves observed. (**E**) 4G2 inhibition of DENV cell infection analyzed using PRNT analysis. Control antibody 9C4 was observed to have no antiviral activity in this assay format.

Using the label-free fusion assay, cross-validated with a live cell microscopy based fusion assay, we examined fusion inhibition of the well-characterized anti-DENV monoclonal antibody (MAb) 4G2^31^. The epitope specificity of this MAb has been mapped to the fusion peptide, which is partially hidden within the mature DENV virion, and becomes fully exposed only during low pH-induced conformational changes within the endosome prior to insertion into the target cell membrane (Fig. 2A)^32^. After confirming the activity of 4G2 in a traditional plaque reduction neutralization (PRNT) assay (Fig. 2E) we examined the anti-fusion activity of 4G2 by both CEI and bright field microscopy (BFM). Using both assay systems, we observed complete inhibition of fusion phenotype following treatment with 4G2 but not an isotype-matched antibody control, 9C4 (both at 500 μg/ml) at the same time as cell exposure to acidified media (Fig. 2B,C). IC_50_ values for both assay systems provided almost identical values (Fig. 2D), confirming the reliability of the label-free system to quantify fusion inhibition for this class of viral fusion proteins. Furthermore, the measurement of CEI immediately proceeding compound treatment and exposure to low pH uncouples any inhibitory effect on syncytia formation from virus replication *per se*. This ensures that any inhibition observed is indeed targeting the fusion process itself and is not simply a consequence of a reduction in virus replication.

### Small compound analysis and structure-activity determination using CEI

**Figure 3:**
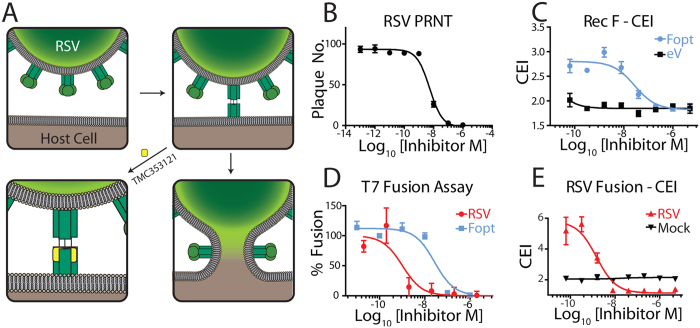
Label-free quantification of RSV F mediated fusion by TMC-353121. (**A**) Schematic of the mode of action of the small molecule RSV fusion inhibitor TMC-353121. Upon activation of the F protein through receptor engagement, an elongated form exposing a trimeric helical bundle is formed. TMC-353121 (yellow) binds to the trimeric helical bundle, thus preventing the collapse of F into the hairpin post-fusion conformation, which drives membrane fusion. (**B**) TMC-353121 is a potent inhibitor of virus infection as demonstrated by PRNT (IC_50_ of 5.8 nM). (**C**) Anti-fusion activity of TMC-353121 against transiently expressed recombinant F measured using cell impedance (blue). No effect on cell impedance of the compound was observed for cells transfected with a noncoding empty vector (eV, black). (**D**) A luciferase reporter based fusion assay demonstrates the anti-fusion activity of TMC-353121 against both live virus (red) and transiently expressed recombinant F protein (blue). (**E**) Antifusion activity of TMC-353121 against RSV can be measured using cell impedance (red). No change in cell impedance following treatment of control cells with the compound was observed (black).

We next examined the RSV fusion protein (F) small molecule inhibitor, TMC-353121^33^, in order to assess the sensitivity of the impedance assay to detect inhibition of syncytia induced by both virus and recombinantly-expressed fusion proteins. TMC-353121 is thought to inhibit fusion by binding to the inner core of the extended intermediate thereby sterically preventing its collapse and formation of the six helical bundle of the final post-fusion hairpin form (Fig. 3A). Before examination on the CEI assay we first confirmed the activity of TMC-353121 by PRNT and obtained an IC50 value of 5.8 nM (Fig. 3B and Table S1). CEI measurements made at 30 h post infection, representing optimal fusion signal prior to cell monolayer destabilization (Supplementary Fig. S3) were used to derive a dose response curve and associated IC_50_ value. Potent inhibition of virus induced cell fusion was observed using both the label-free (Fig. 3E) and traditional cell viability^34^ (Supplementary Fig. S4) and reporter based assay systems (Fig. 3D, T7 driven luciferase assay^29^, Supplementary Fig. S5). A reduction in CEI measurements compared to mock infected controls was observed for compound treated infected cells above the IC_50_ of TMC (Fig. 3E). This difference was most likely a consequence of a reduction in cell metabolism and associated growth for the infected cells, as confirmed by a cell viability (MTT) assay (Supplementary Fig. S4). Significant, but less potent inhibition compared to live virus, was also observed for recombinant F protein induced fusion (Fig. 3C). This was likely to be due to the different levels and kinetics of F expression on infected compared to transiently transfected cells, as well as the additional viral proteins and host cell responses following infection.

**Figure 4:**
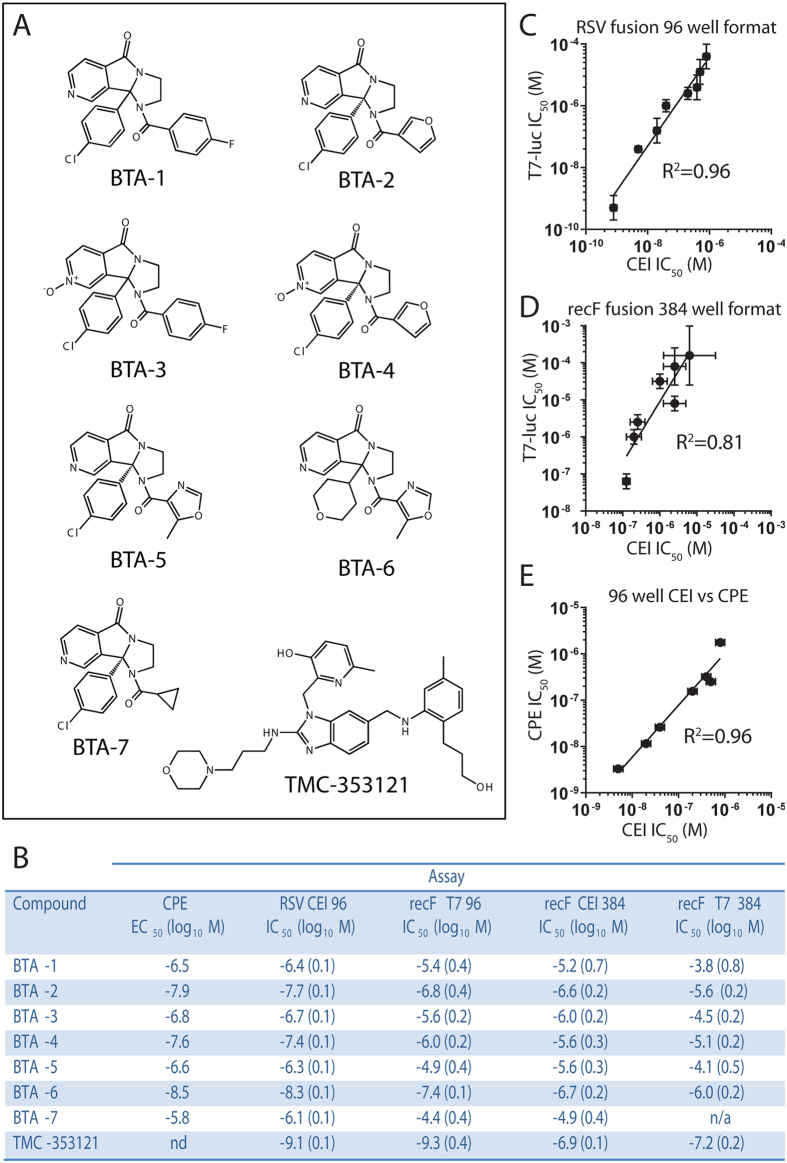
Demonstration of QSAR using a small panel of RSV fusion inhibitors. (**A**) Chemical structures of the inhibitors (BTA-1 to 7 and TMC-353121) used in the assay validation. (**B–D**) Tabular IC_50_ and corresponding scatter plots demonstrating a high correlation between the impedance based assays, whether in 96- or 384-well format with other standard assays over a wide range of inhibitor potencies. Tabulated summary of compound IC_50_ or EC_50_ values (log_10_ M, SE shown in parentheses) measured either by cellular electrical impedance (in 96- and 384-well formats), luciferase reporter cell content mixing assay or a cytopathic effect (CPE) assay. A spread of IC_50_ values over three orders of magnitude was observed using both fusion assays against live virus infection in a 96-well format and a spread of two orders of magnitude is observed for transiently expressed recombinant F (recF) based fusion assays in a 384-well format. The SE reported for the impedance fusion assay is generally smaller than the corresponding luciferase reporter, cell content mixing assay. R^2^ values shown were obtained via linear regression analysis.

We then benchmarked the CEI assay using a panel of small molecule hits against RSV with previously defined inhibition profiles (Fig. 4A, kindly provided by Biota Pharmaceuticals). These compounds were developed as part of a lead optimization strategy targeting the F protein and possessed a range of antiviral activities (nM to μM) for RSV infected cells in culture^35^. Mode of action studies using cell-based syncytium formation assays^30^ confirmed that these compounds were fusion inhibitors.

Cells transiently transfected with the RSV F expression plasmid or infected with RSV were incubated with a dilution series of test compounds in microtiter plates. Concentration-dependent inhibition of fusion-mediated events in both virus-infected and transfected cells was observed. The IC_50_ values determined from the CEI measurements of RSV infected cells were in good agreement with data derived from traditional T7 luciferase and already established cell viability data, across two orders of magnitude of potency (Fig. 4B–E). The potencies of all fusion inhibitors were comparable between RSV infected and recombinant F expressing cells, however the same relative shift in activity seen between RSV-infected and transfected cells, as noted for TMC-353121, was also observed (Fig. 4 B,C,D). This observation further supports the suggestion that the effect was either due to differential levels of F-protein expressed in transfected versus virus infected cultures or difference in the fusion mechanics in the context of additional viral proteins and host cell responses following infection. Successful transfer of the assays to a 384-well format (Fig. 4B,D; R^2^ of 0.81) confirmed that CEI was suitable for screening of fusion inhibitors in a high-throughput format.

Additionally, the confirmation of a fusion signature for recF also raised the possibility of using CEI to probe the molecular basis of the viral fusion process. To demonstrate the applicability of CEI measurements to probing structure-function relationships at the protein level we used site directed mutagenesis to generate two recF constructs that were observed at the microscopic level to either inhibit or enhance fusion activity (Supplementary Fig. 6D). The fusion phenotype was confirmed for both mutants using the impedance assay (Supplementary Fig. S6A–C) indicating that the platform was suitable for interrogating fusion phenotype changes resulting from site-directed mutagenesis analysis.

## Discussion

The molecular processes responsible for entry of a wide range of viruses is a major focus of investigation and constitutes an attractive target for antiviral intervention. To date, the majority of therapeutic strategies have involved targeting key viral enzymes involved in replication and protein processing. A significant hurdle to their effective delivery is the requirement for uptake into the host cell for viral inhibition to take place. Therapeutic strategies targeting the viral receptor and fusion machinery circumvent this problem by inhibiting the virus before it enters the host cell. Furthermore, because these processes are so critical for viral infection, the targets are often well conserved across viral families and therefore may offer opportunities for the development of more generic therapeutics. Central to the investigation of the fusion process and the screening of anti-fusion compounds is a requirement for a reliable and robust assay for membrane fusion.

In this study we have demonstrated the use of a novel CEI based assay that can be used to quantify viral induced cell fusion for representatives from all three viral fusion classes. In addition, we have demonstrated its application in characterizing fusion targeting antiviral antibodies and for the characterization of small molecules that inhibit virus driven membrane fusion. The assay allows for real time analysis that facilitates rapid development of experimental protocols in a range of different virus-cell systems.

In all of the viral systems investigated, the fusion signature observed was positively correlated with CEI measurements. This finding is consistent with the formation of multi-nucleated syncytia and the concomitant reduction in extracellular space. As the CEI measurement can be impacted upon by many cellular and environmental factors it is essential that other factors that can effect CEI be accounted for. Cell death, which is a common outcome of viral infection, results in a reduction in CEI. However, other factors such as cell division and spreading must also be carefully controlled for, to allow accurate interpretation of the fusion signature. Here correlative assays can be useful tools to confirm cell fusion kinetics.

It is also worth considering the mechanisms underlying cell fusion when interpreting the CEI fusion signature. In some viral systems, such as DENV and VSV, fusion can be initiated with the addition of an acidic trigger that results in simultaneous triggering of fusion proteins across the whole monolayer and a rapid fusion signature on the order of minutes. Other viral systems, such as RSV, occur with the build-up of fusion proteins on the cellular surface and a slower profile on the order of hours to days can be expected. While these differences require careful consideration and appropriate control conditions, they also highlight the versatility of CEI in providing label-free and real-time measurements.

These characteristics underpin the utility of the CEI fusion assay which could in theory be applied to any adherent cell line in which syncytia formation is observed, paving the way for fusion assays in more representative cell lines and viruses that are more difficult to culture. Given the broad applicability, reproducibility and ease of use, CEI measurement of cell fusion should provide a powerful new tool in screening for new anti-viral agents and in investigations of membrane fusion for both well-studied and newly emerging enveloped viruses.

## Methods

### Cells and viruses

Vero, Cos7 and BsrT7 cells (the latter kindly provided by Prof K. Conzelmann) were maintained in Opti-MEM media (Invitrogen) supplemented with 4% fetal bovine serum (FBS). Cos7 cells stably expressing either EGFP (Cos7-G) or mCherry (Cos7-R) were clonally selected in the presence of geneticin (Invitrogen) at 1 mg/ml. All stable cell lines were maintained by the addition of 500 μg/ml geneticin to every second passage. BEAS-2B cells were maintained in DMEM (Invitrogen) supplemented with 10% FBS. A549 cells were grown in DMEM/F12 (Invitrogen) with 10% FBS. All mammalian cell lines were incubated at 37 °C in a humidified incubator supplemented with 5% CO_2_. C6/36 cells were grown at 28 °C in RPMI media (Invitrogen) supplemented with 10% FBS and 25 mM HEPES. DENV, stocks were propagated in C6/36 cells. RSV (A2) was grown in A549 cells. HSV-1 and HSV-2 stocks were propagated in vero cells All viral titers were determined by serial dilution on vero cells using an immunoplaque detection method.

### DENV cellular electrical impedance (CEI) fusion assay

A visual overview of the CEI fusion assay is outlined in Fig. 1A. 96-well E-Plates (Roche) were pre-blanked to establish a baseline CEI reading using serum free (SF) RPMI media before seeding with C6/36 cells at a density of 6 × 10^4^ cells/well. Plates were sealed with parafilm and placed into the xCELLigence SP station (Roche) for 24 h within a 28 °C incubator. Cell impedance was recorded every 15 min for every active well. Virus (or mock) in SF media was then added to cells at a multiplicity of infection (MOI) of two. After 2 h post infection the inoculum was replaced with growth media and plates were returned to the SP station for 24 h. At 48 h post cell seeding, the E-plate was removed and the SP station was transferred to a 40 °C incubator to equilibrate. At 50 h post plating, media was removed from cells, replaced with pre-warmed fusion media (HEPES buffered RPMI at pH 7.5, or MES buffered RPMI at pH 6, 40 °C). E-Plates were sealed and returned to the SP station and incubated at 40 °C. CEI measurements were taken every 30 sec for 3 h and every 15 min for an additional 15 h.

### RSV/F CEI fusion assay

Impedance assays were setup in parallel to microscopy based fluorescent protein cytoplasmic mixing and cell content mixing T7 driven fusion assays. For testing small molecule inhibitors see cell content mixing, T7 driven reporter fusion assay protocol for cell infection, transfection and plating method. All other steps were as listed below.

Cos7-G cells were transfected with the RSV F expression vector (pCICO-Fopt [30]) in 6 well plates using lipofectamine LTX as per manufacturer’s instructions (Invitrogen) or infected with RSV (MOI of 2). Four hours post infection/transfection, cells were trypsinized and re-suspended to 8 × 10^5^ cells/ml. E-Plates were pre-blanked and Cos7-G and Cos7-R cells were seeded together at a 1:1 ratio for a final cell density of 5 × 10^4^ cells/well. Plates were placed into the SP station, incubated at 37 °C in 5% CO_2_ and impedance was recorded every 15 min over 72 h.

### VSV CEI fusion assay

Cos7-G cells were transfected with the VSV G protein expression vector (pMD.G [31]). Four hours post transfection cells were trypsinized and re-suspended to 4 × 10^5^ cells/ml. After blanking, media was removed from E-Plates and Cos7-G cells were mixed with Cos7-R at a 1:1 ratio and plated at a final cell density of 5 × 10^4^ cells/well. Plates were placed into the SP station in the 37 °C incubator and impedance was recorded every 15 min. At 24 h post plating, media was removed and pre-warmed fusion media (HEPES buffered RPMI at pH7.5, or MES buffered RPMI at pH5.5) was added to cells. E-Plates were immediately returned to the SP station within the incubator. CEI measurements were taken every 30 sec for 3 h and every 15 min for an additional 15 h.

### DENV live cell bright field microscopy (BFM) fusion assay

Assays were setup as per the DENV CEI fusion assay using clear bottom microtitre plates in place of E-plates. After addition of the fusion media plates were sealed and immediately transferred to the image chamber of the InCell Analyzer 1000 (GE healthcare) which had been pre-warmed to 40 °C. Bright field images were obtained every 10 min for 24 h. Image analysis was performed using the Incell Analyser software (GE healthcare), using the multi-target mode. Cell numbers were used to infer levels of cell fusion. IC_50_ values for anti-fusion compounds were derived from a three-parameter dose response curve fit to a single time point corresponding to maximum fusion within the impedance fusion assay controls.

### RSV/VSV live cell BFM fusion assay

To visualise fusion in mammalian cells two populations of stable Cos-7 cells expressing either GFP (Cos7-G) or mCherry (Cos7-R) were used. Fusion of cells resulted in cytoplasmic mixing and an overlap of GFP and mCherry fluorescent signal (overlap presented in yellow, Fig. 1E,F). Cells were prepared as per the CEI based fusion assay, using clear bottom microtitre plates in place of E-Plates. For visualization of RSV fusion, the cells were incubated within the InCell Analyzer (set at 37 °C) for the entire course of the experiment, post seeding. Images were obtained every 15 mins using bright field illumination and fluorescence imaging. For VSV-G, plates were placed within the InCell Analyzer after the addition of fusion media, as per the DENV live cell microscopy fusion assay and imaged every 10 min for 24 h.

### Cell content mixing T7 fusion assay

An overview of the cell content mixing fusion assay is shown in Fig. S5. The protocol is an adaptation of that described by Bagai and Lamb^29^. Cos7 cells were transfected with a plasmid containing Renilla luciferase under T7 promoter control (pGL80-T7RL) and an RSV F expression vector. For live virus fusion assay, transfection media was removed after 2 h and replaced with SF media containing RSV (MOI of 2). Two h post infection (4 h post initial transfection) cells were trypsinized and re-suspended to 8 × 10^5^ cells/ml. Compounds were serially diluted in SF Opti-MEM media and added to opaque bottom, white microtitre plates (Corning) at 50 μl/well along with 25 μl of both Cos7 and BsrT7 cells added to each well to obtain a final concentration of 5 × 10^4^ cells/well. After 24 h, media was exchanged with 50 μl/well Opti-MEM media supplemented with 2% FBS and 8 ng/ml Enduren Live Cell Substrate (Promega). After 6 h at 37 °C in a humidified incubator supplemented with 5% CO_2_, the plates were read using a Lucy 2 luminometer with 1 sec integration time. Assays were performed in triplicate.

### 384-well adaptation of fusion assays

384-well fusion assays were setup as per 96-well protocol with the following changes: Cos7 and BsrT7 cells were seeded at a final concentration of 1 × 10^4^ cells/well. Compound titration was performed in a Biomek NX Laboratory Automation Workstation and added to a final volume of 25 μl/well. Luciferase activity was determined using an EnVision Multilabel reader (PerkinElmer).

### Plaque-reduction neutralization (PRNT) assay

Confluent monolayers of vero cells grown in 96-well plates were washed with PBS prior to viral infection. Virus was mixed at a 1:1 ratio with a titration of small molecule inhibitor or antibody prior to addition to cells at a final virus concentration of 100 pfu/well. Cells and virus were incubated for 2 h with the addition of a carboxymethyl cellulose (CMC) overlay comprising 1.5% CMC, 2.5% FBS in MEM media (Sigma). Cells were then incubated for 48 h and fixed with ice cold 80% acetone in PBS. Plates were dried and blocked with 5% skim milk powder in PBS supplemented with 0.05% Tween-20 (PBST-SMP). Primary antibody (rabbit anti-F for RSV, 4G2 for DENV, YFV) was added (1:1000 dilution in PBST-SMP) at 50 μl/well. After incubation at 37 °C for 1 h, plates were washed three times with PBST and then incubated with secondary antibody (IRDye800DW donkey anti-rabbit, LI-COR Biosciences). Plates were incubated and washed as above before drying. Plates were scanned on the Odyssey imager (LI-COR Biosciences) at 41 μM resolution and plaques counted by eye with the aid of computer imaging software (Adobe Photoshop). IC_50_ values for PRNT were derived from a three-parameter dose response curve fit to plaque numbers obtained for compound titration performed in triplicate.

### RSV cytopathic effect (CPE) reduction assay

The anti-viral activity of RSV inhibitors was determined using an adaptation of the CPE assay that quantifies the loss of cell viability induced by syncytium formation. HEp-2 cells were trypsinized and re-suspended in MEM supplemented with 2% FBS to a concentration of 1.5 × 10^5^ cells/ml. Virus was mixed with cells at a MOI of 0.2 and cells were seeded into 96 well plates at 1.5 × 10^4^ cells/well. Test compounds were serially titrated and added to cells. Assay plates were incubated at 37 °C and 5% CO_2_ for 48 h after which MTT (3-(4,5-dimethylthiazol-2-yl)-2,5-diphenyltetrazolium bromide) was added to cells at a final concentration of 1 mg/ml and cells were incubated for 2 h. Media was removed and cells solubilized with 100% isopropanol. Absorbance was read at 540 nm with a reference wavelength of 690 nm. The 50% effective concentration (EC_50_) was determined using non-linear regression.

### Cytotoxicity assay

Potential cytotoxic effects of TMC-353121 in combination with RSV infection were quantified using an MTT assay. Assay setup was identical to the impedance RSV fusion assay, using a mixture of Cos7 and BsrT7 cells. At 29 h post plating, MTT at 400 ng/ml was added in SF media (100 μl/well) and incubated with cells for 1 h in a humidified incubator at 37 °C and 5% CO_2_. Media was removed and cells solubilized with the addition of DMSO (50 μl/well). Absorbance was read at 565 nm in a Spectramax Microplate Reader (Molecular Devices).

### Scanning electron microscopy

Cell monolayers on E-Plates were washed with PBS and then fixed with 2.5% glutaraldehyde in PBS and post-fixed with 1% osmium tetroxide. Samples were gradually dehydrated from 20–100% ethanol followed by critical point drying in CO_2_. The bottom of E-Plate wells were prepared, mounted on aluminium stubs and given a conductive coating of platinum. Samples were visualized with a JEOL JSM-6610 SEM with an accelerating voltage of 10 kV.

We wish to thank members of the Young and Cooper laboratories for valuable discussions and help during the conduct of this study. We also thank Biota Pharmaceuticals for the generous supply of the RSV small compound inhibitor series and for the CPE assay data reported herein. The authors also acknowledge the facilities and the scientific and technical assistance of the Australian Microscopy and Microanalysis Research Facility at the Centre for Microscopy and Microanalysis at the University of Queensland.

## Additional Information

**How to cite this article**: Watterson, D. *et al.* A generic screening platform for inhibitors of virus induced cell fusion using cellular electrical impedance. *Sci. Rep.* **6**, 22791; doi: 10.1038/srep22791 (2016).

## Supplementary Material

Supplementary Information
