# Supplementary material for: A generic screening platform for inhibitors of virus induced cell fusion using cellular electrical impedance
Source: Sci Rep. 2016 Mar 15;6:22791. doi: 10.1038/srep22791 (PMC4792136; doi:10.1038/srep22791)
Supplement: Supplementary Information [file srep22791-s1.pdf]

Supplementary Information

A generic screening platform for inhibitors of virus induced cell fusion using cellular electrical impedance

Authors: Daniel Watterson[1,2,3], Jodie Robinson[1], Keith J. Chappell[1,3], Mark S. Butler[2,3], David J. Edwards[2], Scott R. Fry[2], Imogen Bermingham[1,3], Matthew A. Cooper[2,3,*] & Paul R. Young[1,2,3,*]

Affiliations:

[1]School of Chemistry and Molecular Biosciences, University of Queensland, Brisbane, Queensland 4072, Australia

[2]Division of Chemistry and Structural Biology, Institute for Molecular Bioscience, University of Queensland, Brisbane, Queensland 4072, Australia

[3]Australian Infectious Diseases Research Centre, University of Queensland, Brisbane, Queensland 4072, Australia

*To whom correspondence should be addressed:

P.R.Y. (p.young@uq.edu.au) & M.A.C. (m.cooper@uq.edu.au)

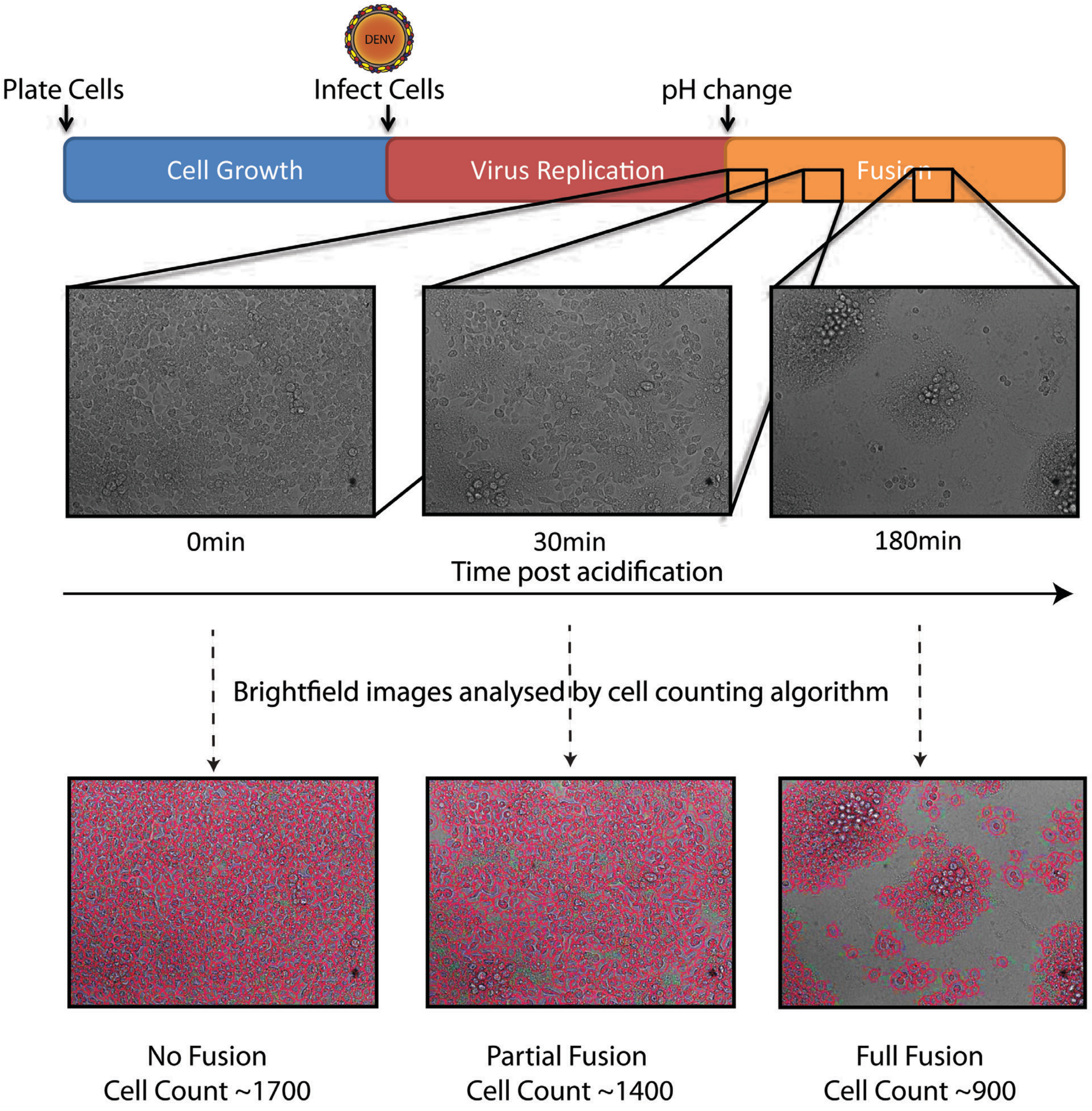

Figure S1 Overview of live cell bright-field microscopy (BFM) fusion assay. Cells are grown and infected with DENV and media is exchanged 24h post infection. Fusion is initiated with acidified buffer (pH6) and bright-field images are obtained every 30s using the InCell analyzer 1000 (GE Healthcare Life Sciences, Australia). Cell number is calculated using Incell Analyzer image analysis software and fusion expressed as a reduction in cell number arising from syncytia formation.

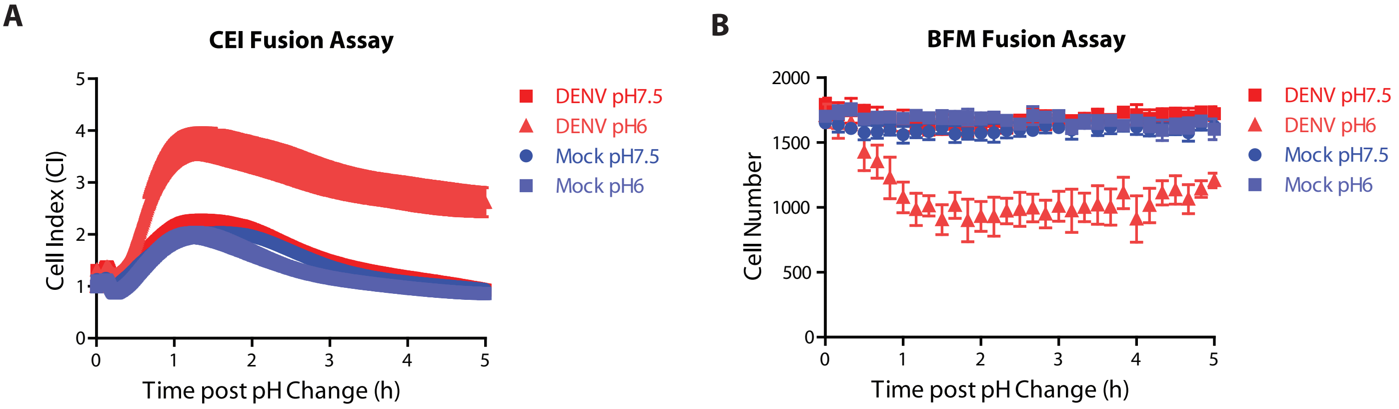

Figure S2 Comparison of assay kinetics. Kinetic data is presented for both (A) CEI and (B) BFM based fusion assays. Data points are shown for both DENV infected and mock infected cells that have been treated with pH adjusted buffered media at 24h post infection. A significant increase in the impedance readout relative to the control samples is observed for DENV infected cells when acidified media is used and peaks approximately 1.5h post addition. Similar kinetics are observed in the live cell microscopy based assay where fusion was measured by a reduction in cell number with peak reduction observed approximately 1.5h post low pH induction.

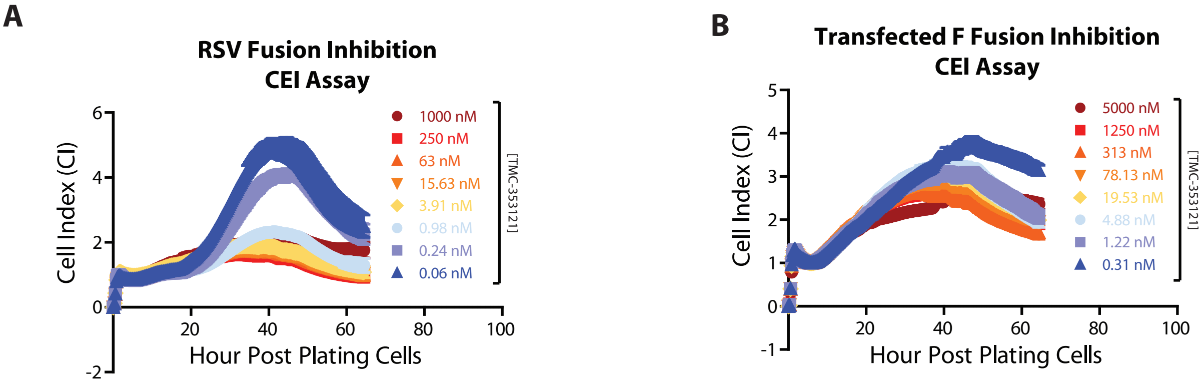

Figure S3 Raw cell impedance data used to calculate TMC-353121 anti-fusion potency. Impedance and clear bottom microtitre plates were seeded in parallel with a mix of BsrT7 cells (a stable cell line constitutively expressing the T7 polymerase) and RSV infected cells or Cos7 cells transfected with an expression plasmid encoding the RSV F protein. These latter cells were also transiently transfected with a plasmid encoding the luciferase reporter gene under T7 promoter control. Compound was added at the time of plating and cell impedance measured every 15mins for the course of the experiment. A dose response was observed, with a reduction in impedance signal in the presence of higher concentrations of TMC-353121. IC50 values were determined using CIs obtained at 30h post infection, corresponding to optimal fusion signal. Luciferase reagent was added to the clear bottom plate at 28h post plating and emitted light recorded at 30h (Fig. 3C and E).

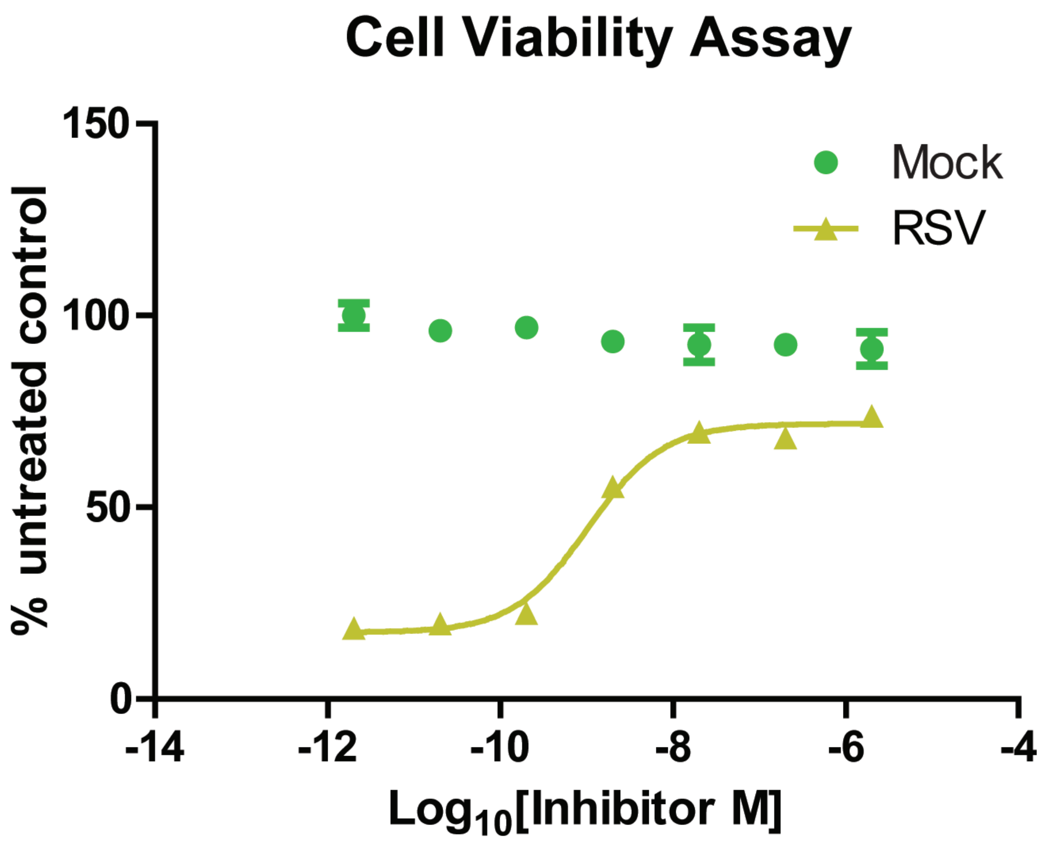

Figure S4 Cell viability of TMC-353121 treated RSV infected cells. An MTT assay was used to determine the viability of RSV infected cells in the presence of TMC-353121. At concentrations above the anti-fusion IC_{50} (determined by impedance assay - Fig 2E) cell viability is seen to plateau at 80% relative to mock infected cells. No reduction in cell viability is observed for mock treated cells at any TMC-353121 concentration indicating that the reduction in impedance values for infected cells beyond the inhibitory effect of the compound is most likely due to viral replication limiting cell metabolism and proliferation.

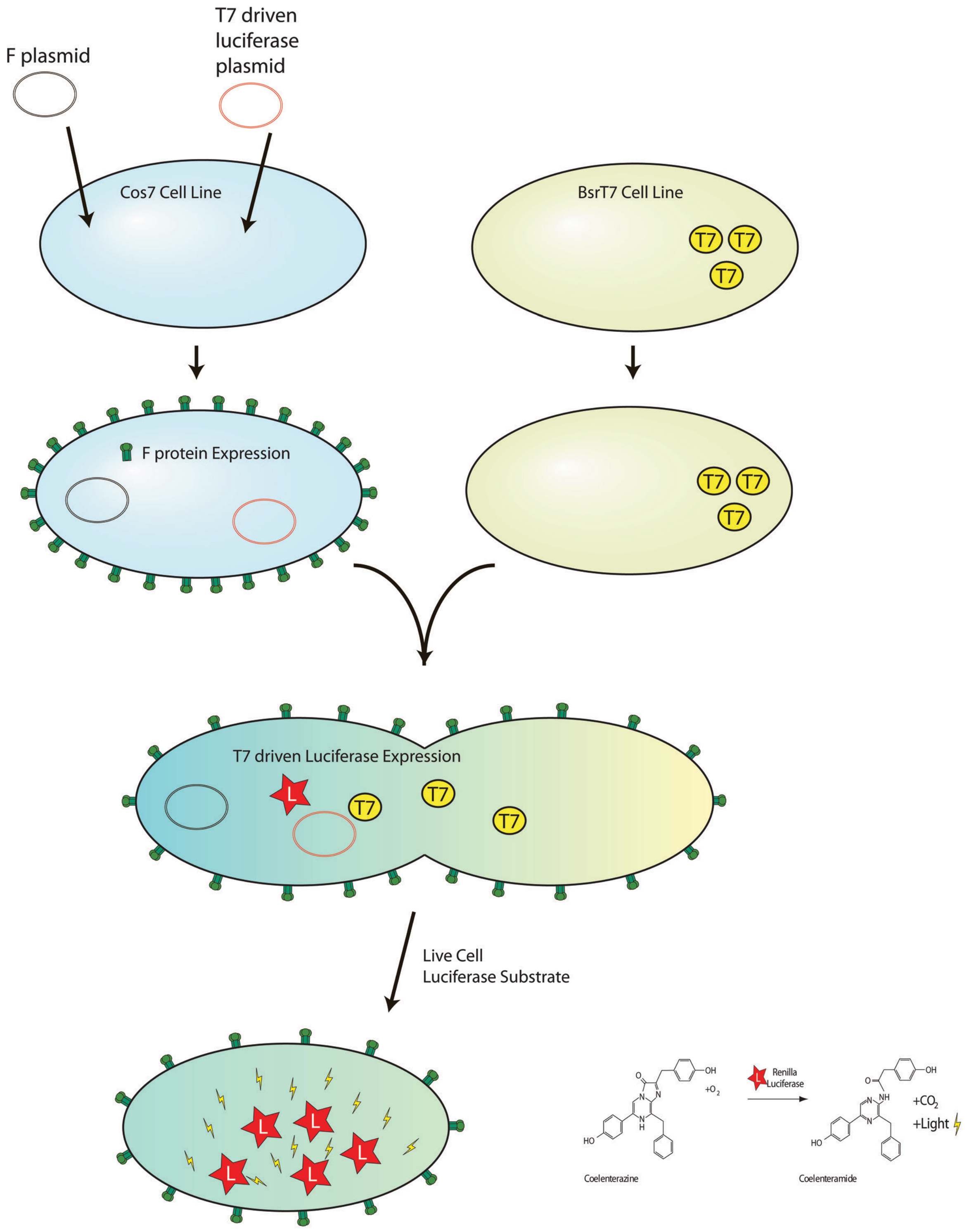

Figure S5 Schematic of the luciferase reporter based, cell content mixing, T7 fusion assay. Cos7 cells are infected or transfected with a fusion protein expression plasmid along with a plasmid encoding a reporter gene (luciferase) under T7 promoter control. After incubation to allow protein expression, cells are mixed with a cell line stably expressing the T7 polymerase (BsrT7). Cell fusion, mediated by the presence of viral fusion proteins, results in cytoplasmic mixing leading to the transcription and translation of the reporter gene. Luciferase substrate is added and relative fusion activity can be measured as a function of luciferase generated light emission.

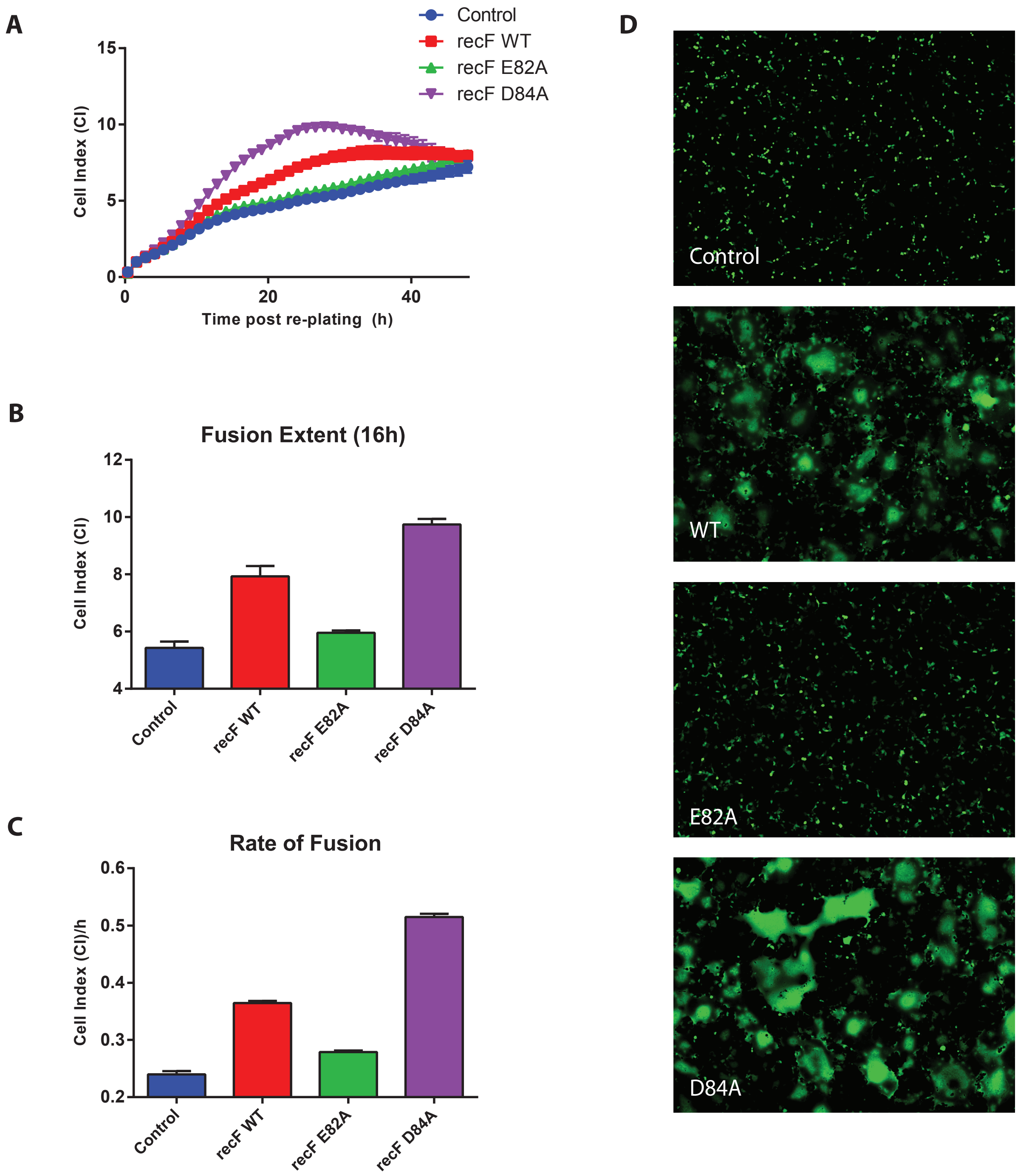

Figure S6 Impedance based fusion assay examination of RSV F mutant proteins. Cos7 cells were transfected with expression plasmids encoding wild-type RSV F protein (recF WT), mutant recF constructs (recF D84A and recF E82A) or empty plasmid as a negative control. 4 hours post transfection cells were plated into E-plates and clear well microscopy plates in parallel. Cell index was measured every 15mins post plating and a clear increase in impedance measurement and rate of increase can be seen for recF WT vs control (A). The E82A and D84A mutants are shown to have decreased and increased fusion function, respectively (B and C). This result was reflected in the fusion phenotype observed using live cell microscopy which visualized transfected cells via the biscistronic expression of EGFP (D).

	Assay	IC_{50} (nM)
DENV 4G2	Impedance	36.4 ± 13.8-96.5
	Microscopy	49.3 ± 20-121.6
	PRNT	164.3 ± 103.3-261.63
RSV TMC-353121	Virus Impedance	1.6 ±0.8-3.3
	Virus T7	1.0 ± 0.3-3.6
	recF Impedance	17.2 ± 5.8-50.6
	recF T7	28.2 ± 12.9-61.5
	PRNT	5.8 ± 4.4-7.7

Table S1 Summary of IC50 values obtained for DENV targetting MAb 4G2 and small molecule RSV fusion inhibtor TMC-353121.
